# Risk of hip fracture in meat-eaters, pescatarians, and vegetarians: results from the UK Women’s Cohort Study

**DOI:** 10.1186/s12916-022-02468-0

**Published:** 2022-08-11

**Authors:** James Webster, Darren C. Greenwood, Janet E. Cade

**Affiliations:** 1 Nutritional Epidemiology Group, School of Food Science and Nutrition, University of Leeds, Leeds, LS2 9JT UK; 2 School of Medicine, University of Leeds, Leeds, LS2 9JT UK

**Keywords:** Diet, Nutrition, Vegetarian, Hip fracture, Cohort study

## Abstract

**Background:**

The risk of hip fracture in women on plant-based diets is unclear. We aimed to investigate the risk of hip fracture in occasional meat-eaters, pescatarians, and vegetarians compared to regular meat-eaters in the UK Women’s Cohort Study and to determine if potential associations between each diet group and hip fracture risk are modified by body mass index (BMI).

**Methods:**

UK women, ages 35–69 years, were classified as regular meat-eaters (≥ 5 servings/week), occasional meat-eaters (< 5 servings/week), pescatarian (ate fish but not meat), or vegetarian (ate neither meat nor fish) based on a validated 217-item food frequency questionnaire completed in 1995–1998. Incident hip fractures were identified via linkage to Hospital Episode Statistics up to March 2019. Cox regression models were used to estimate the associations between each diet group and hip fracture risk over a median follow-up time of 22.3 years.

**Results:**

Amongst 26,318 women, 822 hip fracture cases were observed (556,331 person-years). After adjustment for confounders, vegetarians (HR (95% CI) 1.33 (1.03, 1.71)) but not occasional meat-eaters (1.00 (0.85, 1.18)) or pescatarians (0.97 (0.75, 1.26)) had a greater risk of hip fracture than regular meat-eaters. There was no clear evidence of effect modification by BMI in any diet group (*p*-interaction = 0.3).

**Conclusions:**

Vegetarian women were at a higher risk of hip fracture compared to regular meat-eaters. Further research is needed to confirm this in men and non-European populations and to identify factors responsible for the observed risk difference. Further research exploring the role of BMI and nutrients abundant in animal-sourced foods is recommended.

**Trial registration:**

ClinicalTrials.gov, NCT05081466

**Supplementary Information:**

The online version contains supplementary material available at 10.1186/s12916-022-02468-0.

## Background

Hip fractures are most common in elderly women [1] and are becoming increasingly prevalent in the UK and globally due to growing ageing populations [2, 3]. Health-related quality of life declines after hip fracture and mortality increases [1, 4]. Social and economic costs from hip fractures are also substantial [2], with an international average cost 12 months after the first hip fracture of $44,000 per patient [5]. There are growing concerns regarding bone health and fracture risk in individuals on meat-free diets [6–9], but associations between these diet groups and hip fracture risk remain unclear. An estimated 5% of the US population [10], 3% of the UK population [11, 12], and 30% of India’s population follow vegetarian diets [13]. The number of vegetarians worldwide is increasing [7], possibly due to accumulating evidence of reduced risks of several chronic diseases, including diabetes [14], ischaemic heart disease, and cancer [15], and a lower environmental footprint of vegetarian diets compared to omnivorous diets [16, 17]. Understanding hip fracture risk in vegetarians in particular is therefore becoming increasingly important to public health.

Whilst diet quality varies among vegetarians [16], vegetarian diets are often characterised by a higher intake of fruits and vegetables including foods high in vegetable protein [8], which have been associated with a reduced hip fracture risk in adults in reviews of previous epidemiological studies [18–21]. However, vegetarian diets have also been characterised by lower dietary intakes of nutrients that have been positively associated with bone mineral density (BMD) and are more abundant in animal products than in plants. Examples include total protein, calcium, vitamin D, vitamin B12, and ω-3 fatty acids [6, 22], though associations between these nutrients and hip fracture risk are unclear and complex [20]. Studies have also reported a lower average body mass index (BMI) in vegetarians and pescatarians compared to omnivores [8, 23], which has been inversely associated with hip fracture risk [24]. Risk differences for hip fracture between vegetarians, pescatarians, and meat-eaters are therefore plausible, but evidence is limited in exploring these dietary patterns.

Cross-sectional studies show lower BMD in vegetarians compared to non-vegetarians [25, 26], but prospective studies comparing the risk of hip fracture in these diet groups over time are scarce and limited [8, 9]. The recently published European Prospective Investigation into Cancer (EPIC)-Oxford cohort study of UK men and women showed a greater risk of hip fracture in pescatarians, vegetarians, and vegans compared to meat-eaters [8]. The Adventist Health Study-2 (AHS-2) also showed a greater risk of hip fracture in vegans but not vegetarians compared to meat-eaters in US women, but with outcome data based on self-administered questionnaires [9]. To our knowledge, no other prospective study has compared the risk of hip fracture in vegetarians and non-vegetarians; therefore, associations between these diet groups and hip fracture risk require further investigation.

The United Kingdom Women’s Cohort Study (UKWCS) has been enriched with vegetarians and pescatarians, so is well-suited to study the risk of chronic diseases over time in these diet groups [27]. Our objectives were therefore to investigate the risk of hip fracture in occasional meat-eaters, pescatarians, and vegetarians compared to regular meat-eaters in middle-aged UK women and to determine if potential associations between each diet group and hip fracture risk are modified by BMI.

## Methods

We followed the Strengthening the Reporting of Observational Studies in Epidemiology – Nutritional Epidemiology (STROBE-nut) guidelines for the reporting of cohort studies (Additional file 1: Table S1) [28].

### Study design and participants

The UKWCS has been described in detail elsewhere [27]. In brief, 500,000 women from England, Scotland, and Wales responded to a direct mail questionnaire from the World Cancer Research Fund (WCRF) between 1995 and 1998. Of the 75% that agreed to participate in a more detailed survey, those who identified as vegetarian or non-red meat-eaters, and were aged 35–69 years when completing the WCRF questionnaire, were eligible for inclusion in the UKWCS. For each vegetarian, the next non-vegetarian or red meat-eater who was aged within 10 years of the vegetarian was selected to form a comparison group. In total, 35,372 women across the UK, aged 35–69 years, responded to a postal questionnaire that collected dietary, lifestyle, demographic, and anthropometric data at recruitment (1995–1998). This approach was taken to maximise power in comparing the risk of hip fracture across the diet groups [29]. Participants were then excluded if they lived outside of England (*n* = 3821), had a hip fracture on or before the date of recruitment according to hospital episode statistics (*n* = 2), had missing age data (*n* = 364), or had outlier FFQ or covariate data (daily energy intake < 500 kcal or > 5000 kcal, BMI < 10 or > 60 kg/m^2^, or FFQ intakes > 3 standard deviations from the mean; *n* = 941), leaving 30,244 participants potentially eligible for inclusion in this study (Additional file 1: Fig. S1). Ethical approval was granted from the National Research Ethics Service Committee for Yorkshire & the Humber – Leeds East (reference 15/YH/0027) at the cohort’s inception in 1993 and was updated to include linkage outcomes, such as hip fracture incidence, in 2017 (reference 17/YH/0144).

### Diet group

Dietary habits of cohort participants over 12 months were assessed at recruitment using a self-administered 217-item food frequency questionnaire (FFQ). The FFQ was validated by comparison with 4-day weighed food diaries and a repeated FFQ on 283 women, both administered 3 years after baseline [27]. Based on the responses to questions on meat, fish, eggs, and dairy intakes, participants were classified as regular meat-eaters (ate meat ≥ 5 times/week), occasional meat-eaters (ate meat < 5 times/week), pescatarians (ate fish but not meat), vegetarians (ate eggs or dairy but not meat or fish), or vegans (did not eat meat, fish, eggs, or dairy). Vegans were combined with the vegetarian group due to the small number of vegan participants (*n* = 130) and cases (*n* = 5). Participants with intakes of a food item of less than once per month were considered non-consumers. Further details on the questionnaire and classification of diet groups are provided in Additional file 1: Supplementary methods and Table S2.

### Outcome

Participants’ diet and lifestyle characteristics were linked with their hospital episode statistics up to 31 March 2019. The primary outcome was hip fracture incidence (International Classification of Diseases, ICD-9 code 820, ICD-10 codes S72.0–72.2, Table S3). We also used hip replacements (ICD-10 code Z96.64) as an indicator of hip fracture, but no additional cases were identified using these criteria. The time frame was person-years until hip fracture incidence, or until the end of the study period or death in non-cases, using attained age as the timescale [30].

### Statistical analysis

All statistical methods were registered in advance on ClinicalTrials.gov (NCT05081466).

Socio-demographic, lifestyle, anthropometric, and nutritional characteristics of UKWCS participants at recruitment were summarised by diet group using descriptive statistics. Cox proportional hazard regression models were fitted to estimate hazard ratios (HR) and 95% confidence intervals (95% CI) for the associations between each diet group and hip fracture risk, with regular meat-eaters as the reference group. The target estimand was the relative causal effect of each diet group on hip fracture risk compared to regular meat-eaters. Cox models used weights based on the inverse probability of being sampled to account for the over-sampling of pescatarians and vegetarians at recruitment, increasing the representativeness of the cohort to the UK population [29].

We applied both unadjusted and multivariable-adjusted models. Both models controlled for age by using attained age as the timescale [30]. Additional confounders included in the adjusted model were based on a directed acyclic graph (DAG), following available guidelines on their creation and reporting [31], and included (all at recruitment): ethnicity (white, Asian, black, other); socio-economic status (SES, professional/managerial, intermediate, routine/manual); marital status (married/living as married, separated/divorced, single/widowed); menopausal status (premenopausal, postmenopausal); number of children (continuous); prevalence of cardiovascular disease, cancer, or diabetes at recruitment (yes, no); physical activity in hours per day (continuous); smoking status (current, former, never); alcohol consumption (> 1/week, ≤ 1/week, never); BMI (continuous); and any nutritional supplement use (yes, no). The DAG and definitions of confounders are given in Additional file 1: Supplementary methods, Table S3, and Fig. S2. The proportional hazards assumption was assessed based on Schoenfeld residuals and was not violated for all terms in the adjusted model.

To determine the role of BMI as a potential effect modifier, we added dichotomized BMI level (< 23.5, ≥ 23.5 kg/m^2^) to the adjusted model as an interaction term with each diet group, with these cut-off points defined to ensure a similar number of participants in each stratum. We also added individual BMI (continuous per kg/m^2^ increase) to the adjusted model as an interaction term with each diet group and omitted BMI from the adjustment set for that analysis. Further exploratory analyses included testing for interaction effects with each diet group for menopausal status (premenopausal, postmenopausal), physical activity level (< 150 minutes/week, ≥ 150 min/week), age (≤ 60, > 60 years), SES (routine/manual, intermediate, professional/managerial), smoking status (current, former, never), and use of any nutritional supplements (yes, no). In each exploratory subgroup analysis, the potential effect modifier was omitted from the relevant adjustment set.

We explored the effect of potential mediators by further adjusting the adjusted model for each mediator independently. Potential mediators were total energy intake and intake of protein, calcium, vitamin D, vitamin B12, monounsaturated fatty acids (MUFA), polyunsaturated fatty acids (PUFA), and zinc from dietary sources only (not including supplemental sources). An adjusted model with BMI removed from the adjustment set is also presented to determine its influence on any associations.

As a sensitivity analysis, we explored the risk of hip fracture in vegans compared to meat-eaters by fitting the adjusted model with vegetarians and vegans separated. Additional sensitivity analyses were as follows: excluding participants with a survival time < 5 years to check for reverse causation, excluding participants on long-term treatment for illness, and further adjusting for hormone replacement therapy (HRT) and prevalence of fracture at sites other than the hip at recruitment (identified in hospital episode statistics), respectively, since these are known risk factors for hip fracture [32]. Participants with missing data for a variable required in a given analysis were excluded from that analysis. We did not impute missing covariate data. All statistical analyses were performed using Stata (version 17).

## Results

### Participants

Of the 30,244 women potentially eligible at recruitment, those with missing covariate data for body weight (*n* = 596), height (*n* = 649), ethnicity (*n* = 811), physical activity (*n* = 1561), marital status (*n* = 460), SES (*n* = 331), or menopausal status (*n* = 309) were excluded, leaving 26,318 women for unadjusted and adjusted analyses. The study flow chart is given in Additional file 1: Fig. S1.

### Descriptive data

Characteristics of the 26,318 cohort participants at recruitment are summarised by diet group in Table 1. Over a median follow-up time of 22.3 years, 822 hip fracture cases were observed (556,331 person-years), corresponding to 3.1% of the cohort. On average, at recruitment, pescatarians and vegetarians were younger than regular meat-eaters, reported higher education levels, were more likely to have professional or managerial jobs and less likely to have routine or manual jobs, and were less likely to be married or have any children. BMI was lower in vegetarians (mean (standard deviation, SD) 23.3 (3.9 kg/m^2^)) and pescatarians (23.3 (3.5 kg/m^2^)) than in regular meat-eaters (25.2 (4.4 kg/m^2^)). Prevalence of CVD, cancer, or diabetes at recruitment was highest in regular meat-eaters (*n* = 1250 (10.2%)), and lowest in vegetarians (222 (5.8%)). Exercise and smoking habits were similar across diet groups, but a higher proportion of vegetarians reported never drinking alcohol than all other diet groups. Regular meat-eaters reported the highest absolute dietary intakes of protein, vitamin D, and vitamin B12, whilst vegetarians reported the lowest. Calcium intakes were similar across the diet groups. Other food and nutrient intakes in each diet group are summarised in Additional file 1: Table S4. Characteristics of the cohort at recruitment were similar when including or restricting to participants with missing covariate data (Additional file 1: Table S5).

**Table 1:** Characteristics of 26,318 UKWCS participants at recruitment by diet group

Characteristics, mean (SD) or ***n*** (%)	Total	Diet group			
		Regular meat-eater	Occasional meat-eater	Pescatarian	Vegetarian
Participants (%)	26,318	13,984 (46.2)	8000 (26.5)	3867 (12.8)	4393 (14.5)
Cases (%)	822 (3.1)	394 (3.2)	247 (3.6)	80 (2.4)	101 (2.6)
**Socio-demographics**					
Age, years (SD)	52.1 (9.2)	53.3 (9.2)	53.2 (9.4)	49.7 (8.5)	48.3 (8.2)
Degree-level education (%)	6502 (26.8)	2306 (20.7)	1780 (28.2)	1143 (35.9)	1273 (35.1)
** Socioeconomic status (%)**					
Professional or managerial	19,057 (72.4)	8518 (69.7)	5120 (74.2)	2576 (76.3)	2843 (74.5)
Intermediate	2440 (9.3)	1117 (9.1)	694 (10.1)	270 (8.0)	359 (9.4)
Routine or manual	4821 (18.3)	2586 (21.2)	1088 (15.8)	531 (15.7)	616 (16.1)
Married (%)	20,268 (77.0)	10,103 (82.7)	5007 (72.5)	2432 (72.0)	2726 (71.4)
White ethnicity (%)	25,992 (98.8)	12,139 (99.3)	6820 (98.8)	3331 (98.6)	3702 (97.0)
**Lifestyle**					
Exercise (h/day)	0.2 (0.5)	0.2 (0.5)	0.2 (0.4)	0.3 (0.5)	0.3 (0.5)
Smoking status (%)					
Current	3513 (13.3)	1678 (13.7)	921 (13.3)	448 (13.3)	466 (12.2)
Former	7947 (30.2)	3519 (28.8)	2078 (30.1)	1161 (34.4)	1189 (31.1)
Never	3110 (11.8)	7024 (57.5)	3903 (56.5)	1768 (52.4)	2163 (56.7)
Alcohol consumption (%)					
> 1 serving/week	13,918 (52.9)	6798 (55.6)	3548 (51.4)	1830 (54.2)	1742 (45.6)
≤ 1 serving/week	9290 (35.3)	4276 (35.0)	2471 (35.8)	1145 (33.9)	1398 (36.6)
Never	3110 (11.8)	1147 (9.4)	883 (12.8)	402 (11.9)	678 (17.8)
Nutritional supplementation (%)	14,009 (53.2)	5902 (48.3)	3881 (56.2)	2070 (61.3)	2156 (56.5)
**Anthropometrics**					
BMI, kg/m^2^ (SD)	24.4 (4.2)	25.3 (4.5)	24.1 (3.9)	23.3 (3.5)	23.3 (3.9)
Height, m (SD)	1.6 (0.1)	1.6 (0.1)	1.6 (0.1)	1.6 (0.1)	1.6 (0.1)
**Dietary nutrient intakes**					
Energy intake, kcal/day (SD)	2300 (654.8)	2445 (640.3)	2069 (605.9)	2294 (656.3)	2259 (658.2)
Protein intake, g/day (SD)	88.1 (26.3)	100.9 (24.8)	77.5 (21.3)	79.6 (23.1)	74.0 (22.3)
≥ 0.75 g protein/kg body weight/day (%)	24,837 (94.4)	12,067 (98.7)	6262 (90.7)	3116 (92.3)	3392 (88.8)
Calcium intake, mg/day (SD)	1135 (365.4)	1163 (344.6)	1060 (356.3)	1183 (395.0)	1138 (398.0)
Vitamin D intake, μg/day (SD)	3.1 (1.7)	3.6 (1.6)	2.9 (1.6)	3.1 (1.8)	1.9 (1.1)
Vitamin B12 intake, μg/day (SD)	2.5 (1.2)	7.5 (2.9)	5.1 (2.2)	4.3 (2.0)	2.5 (1.2)
**Others**					
Premenopausal (%)	11,707 (44.5)	7521 (61.5)	4114 (59.6)	1531 (45.3)	1445 (37.8)
Postmenopausal (%)	14,611 (55.5)	4700 (38.5)	2788 (40.4)	1846 (54.7)	2373 (62.2)
≥ 1 children (%)	20,723 (78.7)	10,263 (84.0)	5324 (77.1)	2468 (73.1)	2668 (69.9)
Prevalence of CVD, cancer, or diabetes (%)	2388 (9.1)	1250 (10.2)	664 (9.6)	252 (7.5)	222 (5.8)

*SD* standard deviation, *SES* social economic status, *BMI* body mass index

### Diet groups

**Fig. 1:**
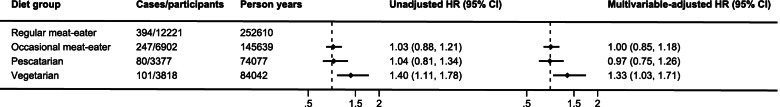
Risk of hip fracture in occasional meat-eaters, pescatarians, and vegetarians compared to regular meat-eaters in the UKWCS. The multivariable-adjusted model was adjusted for the following (all at recruitment): ethnicity (white, Asian, black, other); socio-economic status (professional/managerial, intermediate, routine/manual); marital status (married/living as married, separated/divorced, single/widowed); menopausal status (premenopausal, postmenopausal); number of children (continuous); prevalence of cardiovascular disease, cancer, or diabetes (yes, no); physical activity in hours per day (continuous); smoking status (current, former, never); alcohol consumption (> 1/week, ≤ 1/week, never); BMI (continuous); and any nutritional supplement use (yes, no). HR (95% CI), hazard ratio (95% confidence interval)

Compared with regular meat-eaters, vegetarians (HR 1.40 (95% CI 1.11, 1.78)) but not occasional meat-eaters (1.03 (0.88, 1.21)) or pescatarians (1.04 (0.81, 1.34)) had a greater risk of hip fracture in the unadjusted model (Fig. 1). Adjustment for confounders slightly attenuated these associations in the adjusted model, but the higher risk in vegetarians remained and was statistically significant (vegetarians 1.33 (1.03, 1.71); occasional meat-eaters 1.00 (0.85, 1.18); pescatarians 0.97 (0.75, 1.26)).

### Subgroup analyses

Whilst the risk of hip fracture was 46% higher in participants with BMI < 23.5 kg/m^2^ compared to BMI ≥ 23.5 kg/m^2^, there was no evidence of effect modification by BMI on hip fracture risk in each diet group when BMI was modelled categorically (*p*-interaction = 0.3) or linearly (*p*-interaction = 0.6) (Table 2). There was also no evidence of effect modification in any diet group by age, physical activity, nutritional supplementation, SES, or smoking status (Additional file 1: Table S6). There was some evidence of effect modification by menopausal status, where occasional meat-eaters were at a reduced risk of hip fracture in premenopausal women only (0.43 (0.21, 0.86), *p*-interaction = 0.05).

**Table 2:** Risk of hip fracture in occasional meat-eaters, pescatarians, and vegetarians compared to regular meat-eaters by BMI in the UKWCS

Stratifying variable	***n*** cases/participants, adjusted HR (95% CI)				
BMI		< 23.5 kg/m^**2**^		≥ 23.5 kg/m^**2**^	***p*** interaction
Regular meat-eaters (reference)	161/4927	1.00	233/7294	1.00	
Occasional meat-eaters	123/3554	0.96 (0.75, 1.21)	124/3348	1.05 (0.84, 1.31)	
Pescatarians	50/2075	0.90 (0.65, 1.25)	30/1302	1.06 (0.71, 1.60)	
Vegetarians	72/2338	1.49 (1.10, 2.03)	29/1480	1.02 (0.66, 1.58)	0.3

Models were adjusted for the following (all at recruitment): ethnicity (white, Asian, black, others); socio-economic status (SES, professional/managerial, intermediate, routine/manual); marital status (married/living as married, separated/divorced, single/widowed); menopausal status (premenopausal, postmenopausal); number of children (continuous); prevalence of cardiovascular disease, cancer, or diabetes (yes, no); physical activity in hours per day (continuous); smoking status (current, former, never); alcohol consumption (> 1/week, ≤ 1/week, never); BMI (continuous), and any nutritional supplement use (yes, no)

*HR (95% CI)* hazard ratio (95% confidence interval), *BMI* body mass index

### Sensitivity analyses

The risk of hip fracture appeared higher without adjustment for BMI in vegetarians (1.43 (1.12, 1.83); Table 3). Further adjusting the adjusted model for dietary vitamin D intake in vegetarians increased the magnitude of the association with hip fracture risk (1.44 (1.10, 1.87)), whilst further adjustment for dietary MUFA intake increased the strength of associations for occasional meat-eaters (1.07, 0.90, 1.27)) and vegetarians (1.39 (1.08, 1.79)) (Table 3). Further adjustment for total energy intake and dietary intake of protein, calcium, vitamin B12, and PUFAs did not alter the results substantially.

**Table 3:** Risk of hip fracture in occasional meat-eaters, pescatarians, and vegetarians compared to regular meat-eaters in the UKWCS, with varying levels of adjustment

Model ± further adjustments	HR (95% CI) per diet group			
	Regular meat-eaters (reference)	Occasional meat-eaters	Pescatarians	Vegetarians
Model 1^a^	1.00	1.03 (0.88, 1.21)	1.04 (0.81, 1.34)	1.40 (1.11, 1.78)
Model 2^b^	1.00	1.00 (0.85, 1.18)	0.97 (0.75, 1.26)	1.33 (1.03, 1.71)
Model 2 − BMI	1.00	1.05 (0.89, 1.24)	1.05 (0.81, 1.35)	1.43 (1.12, 1.83)
**Potential mediators**	1.00			
Model 2 + total energy	1.00	1.05 (0.89, 1.24)	0.99 (0.76, 1.28)	1.36 (1.06, 1.75)
Model 2 + dietary protein	1.00	1.03 (0.86, 1.23)	0.99 (0.76, 1.29)	1.36 (1.05, 1.78)
Model 2 + dietary calcium	1.00	1.01 (0.86, 1.20)	0.97 (0.75, 1.25)	1.33 (1.04, 1.72)
Model 2 + dietary vitamin D	1.00	1.03 (0.87, 1.22)	0.99 (0.77, 1.29)	1.44 (1.10, 1.87)
Model 2 + dietary vitamin B12	1.00	1.01 (0.85, 1.22)	0.98 (0.75, 1.30)	1.36 (1.01, 1.82)
Model 2 + dietary MUFA	1.00	1.07 (0.90, 1.27)	1.00 (0.77, 1.30)	1.39 (1.08, 1.79)
Model 2 + dietary PUFA	1.00	1.03 (0.87, 1.21)	0.96 (0.74, 1.24)	1.32 (1.02, 1.69)
Model 2 + dietary zinc	1.00	1.01 (0.85, 1.21)	0.97 (0.75, 1.27)	1.34 (1.03, 1.73)

*HR (95% CI)* hazard ratio (95% confidence interval), *MUFA* monounsaturated fatty acids, *PUFA* polyunsaturated fatty acids

^a^Model 1 included 26,318 participants and was unadjusted

^b^Model 2 included 26,318 participants and was adjusted for ethnicity (white, Asian, black, other), socio-economic status (SES, professional/managerial, intermediate, routine/manual), marital status (married/living as married, separated/divorced, single/widowed), menopausal status (premenopausal, postmenopausal), number of children (continuous), chronic disease prevalence at baseline (yes, no—including stroke, cancer, or diabetes), physical activity in hours per day (continuous), smoking status (current, former, never), alcohol consumption (> 1/week, ≤ 1/week, never), body mass index (BMI, continuous), and any nutritional supplement use (yes, no). All other models were based on the 26,318 participants in model 2

All adjusted results were robust to the addition or removal of other individual covariates from the model, with estimates remaining broadly unchanged across most sensitivity analyses (Additional file 1: Table S7). Exclusion of participants on long-term treatment for illness slightly increased the magnitude of associations in all diet groups (occasional meat-eaters 1.09 (0.88, 1.37); pescatarians 1.08 (0.78, 1.50); vegetarians:1.48 (1.07, 2.04)). Considering vegetarians (96 cases/3688 participants) and vegans (5 cases/130 participants) separately did not substantially alter the estimates in vegetarians (vegetarians 1.38 (1.07, 1.78) and vegans 1.10 (0.42, 2.84)).

## Discussion

### Principal findings

Vegetarians but not occasional meat-eaters or pescatarians were at a higher risk of hip fracture than regular meat-eaters in this cohort of UK women. There was no clear evidence of effect modification by BMI across diet groups. The risk differences remained after accounting for confounders and were not explained by differences in key nutrient intakes related to bone health between vegetarians and regular meat-eaters, implying the potential importance of other unaccounted factors.

### Comparison with previous studies

Prospective evidence of hip fracture risk in individuals on meat-free diets is limited. Our findings largely concur with the results of the only other two cohort studies on this topic [8, 9], strengthening the evidence of a higher risk of hip fracture in UK vegetarian women.

In the EPIC-Oxford cohort, there was evidence of a higher risk of hip fracture in vegetarian women of a similar magnitude (25%) [8]. The slightly higher effect estimate in our study (33%) may be due to our reference group being regular meat-eaters, whereas the reference group in the EPIC-Oxford cohort was meat-eaters of any amount. The AHS-2 also found limited evidence of a 17% higher risk of hip fracture in US vegetarian women [9]. Differences in estimates between the AHS-2 and our results may be due to the different adjustment strategies when accounting for confounders; in the AHS-2, attained age was used as the time frame, and adjustment was made for age and energy, calcium, potassium, and vitamin D intakes at recruitment amongst other factors. This may have resulted in overadjustment and adjustment for factors potentially on the causal pathway, diluting risk estimates. The AHS-2 also relied on self-report for case ascertainment. We identified hip fracture cases using participants’ hospital episode statistics, which incurs less reporting error and selective loss to follow-up. We found no clear evidence of a difference in hip fracture risk in pescatarians or occasional meat-eaters (ate meat < 5 times/week) compared to regular meat-eaters. Similarly, in the AHS-2, there was no clear evidence of a difference in hip fracture risk in semi-vegetarian (ate meat or fish ≤ once/week) or pescatarian women compared to non-vegetarians [9]. In contrast, the EPIC-Oxford cohort study found a 30% increased risk in pescatarian women, potentially due to population differences between EPIC-Oxford and the UKWCS, different intakes of fish or other dietary components, or other sources of residual confounding in either study [8]. Both the EPIC-Oxford and AHS-2 cohort studies reported higher risks of hip fracture in vegans compared to meat-eaters [8, 9]. Due to the low number of vegans in the UKWCS, we could not precisely estimate their risk of hip fracture separate from the vegetarian group. Since vegans may face greater challenges in achieving adequate intake of several nutrients, in particular protein and calcium [6], cohort studies with a high proportion of vegans are needed investigating their risk of hip fracture.

Other epidemiological studies have found that adherence to diets low in meat consumption, such as the Mediterranean diet and Alternative Healthy Eating Index, was protectively associated with hip fracture risk [33, 34], and adherence to Western diets in which meat consumption is high was positively associated with hip fracture risk [35]. Conversely, total meat intake has been inversely associated with hip fracture risk [21]. These results cannot be fairly compared with risks in vegetarians and non-vegetarians, which no other study has directly assessed.

### Interpretation and implications

The observed higher risk of hip fracture in vegetarians compared to regular meat-eaters may be partly explained by the differences in body anthropometrics between the diet groups. Whilst there was no clear evidence of BMI modifying associations between diet groups and hip fracture risk, the lower mean BMI in vegetarians partly explained their higher risk. Previous studies have shown BMI and body weight to be lower in vegetarians [26, 36], and inversely associated with hip fracture risk [24, 37]. Possible mechanisms include the protective roles of bone mass, fat mass, and muscle mass, which have each been inversely associated with hip fracture risk independently [38]. Inadequate fat mass may reduce cushioning from impact force at the hip during falls, which account for 90% of hip fractures [39]. Higher fat mass could also increase bone strength through increased mechanical loading and enhanced oestrogen production [38]. Low muscle mass and strength of the hip flexor muscles and spine extensors have also been associated with an increased risk of hip fracture [40], possibly due to reduced balance and mobility. Weight management may therefore be an important consideration in reducing hip fracture risk in vegetarians, but further research is required to explore the roles of BMI and body composition in hip fracture risk in vegetarians and meat-eaters.

A second potential reason for the higher risk of hip fracture in vegetarians is their lower intake of nutrients important to bone health that are abundant in animal products. Previous studies have found lower dietary intakes of protein, calcium, vitamin D, and vitamin B12 in vegetarians [6, 22], and have suggested protective associations of these nutrients with hip fracture risk [6, 41, 42]. In our study, vegetarians had lower dietary intakes of protein, vitamin D, and vitamin B12, but similar dietary calcium intakes to other diet groups. In particular, vegetarians were less likely to meet the UK recommendation for protein intake in adults of 0.75 g/kg body weight/day than regular meat-eaters (88.8% vs 98.3%) [43], but the higher risk of hip fracture in vegetarians was not explained by any dietary nutrient intake. It is likely that measurement error incurred by estimating nutrient intakes from an FFQ precluded accurate estimation of the importance of nutrients from dietary sources to hip fracture risk in vegetarians.

Since the higher risk of hip fracture in vegetarians remained after adjustment for BMI and several dietary nutrient intakes, other factors may be important. Supplemental sources of specific nutrients and circulating vitamin D concentrations could differ between vegetarians and non-vegetarians and may impact the risk of hip fracture [9, 44] but could not be accounted for in this analysis due to a lack of data. Circulating levels of insulin-like growth factor-1 (IGF-1) may also be lower in vegetarians than in non-vegetarians [45] and have been positively associated with BMD and negatively associated with risk of total fracture and hip fracture [46], but could not be considered here. Future studies should investigate the roles of IGF-1 and nutrients abundant in animal products on hip fracture risk in vegetarians to better understand the reasons for their observed higher risk.

### Strengths and limitations

This study has three main strengths. Firstly, the large number of pescatarians and vegetarians included gave good statistical power to estimate their risk of hip fracture. Secondly, the identification of hip fractures based on hospital records over a long follow-up period reduced reporting errors and loss to follow-up. Finally, we classified subjects into diet groups based on reported intakes of animal foods using a validated FFQ, which may more accurately allocate participants into diet groups than asking participants to identify their diet group.

On average, UKWCS participants were younger by end of follow-up than the average age at hip fracture in women (83 years) [47], limiting the number of hip fractures observed. Moreover, high-energy trauma may account for more hip fractures in younger adults, whereas fragility hip fractures are more common in older adults [48]. We could not distinguish between traumatic and fragility fractures here since information on the cause of hip fractures was not available. We had insufficient power to detect effect modification by covariates in subgroup analyses. For BMI, the strong correlation with the diet group meant that the number of vegetarians with a high BMI or regular meat-eaters with a low BMI was low. Moreover, BMI was derived from self-reported height and body weight, implying a possible measurement error. Investigation of hip fracture risk in underweight participants by diet group was also not possible but merits further investigation.

Women with missing covariate data (*n* = 3926) were excluded from the analyses in this study, which introduced a risk of selection bias. However, the magnitude of any selection bias is unlikely to be clinically significant, given that the characteristics of participants included or excluded in analyses here at recruitment were similar (Additional file 1: Table S5). Although we adjusted for likely confounders, residual confounding was possible. For example, we could not adjust for the use of medications that could impact the associations between the diet groups and hip fracture risk due to a lack of data. The risk of hip fracture could differ between moderate and heavy consumers of alcohol [20], but we were unable to differentiate between these groups when adjusting for alcohol consumption. In addition, the exclusion of participants with prior hip fractures was likely an incomplete exclusion, since hospital data of fracture incidences before 1997 was not available, and the questionnaire did not ask about fracture history. The single questionnaire administered at recruitment was the only method of assessing diet and lifestyle information; therefore, we could not account for changes in the diet group or covariates over time. Additionally, food and nutrient intake in vegetarians in recent years could differ from when data were collected at recruitment due to changes over the last two decades in the availability of vegetarian food products, such as increases in the number of available meat substitute products [49]. Consequently, the generalisability of our findings to modern-day vegetarians is reduced. Our findings were also predominantly in white UK women; previous studies have shown that total fracture risk could depend on ethnicity [50]; therefore, more research is needed investigating hip fracture risk in non-European vegetarians and non-vegetarians.

## Conclusion

Overall, vegetarians but not occasional meat-eaters or pescatarians were at a higher risk of hip fracture compared to regular meat-eaters in this cohort of UK women. Further research is needed to confirm this in other populations, such as men and non-European populations, and to identify the factors responsible for the observed risk difference. In particular, further research exploring the roles of BMI and nutrients abundant in animal-sourced foods is recommended so that public health interventions and policy guidelines aiming to reduce hip fracture risk in vegetarians through dietary change or weight management can be formed.

## Supplementary Information

**Additional file 1: Figure S1.** Flow chart of UKWCS participants. **Figure S2.** Directed Acyclic Graph showing the relationship between diet group, hip fracture incidence, and related factors. **Table S1:** Strengthening the reporting of observational studies in nutritional epidemiology (STROBE-Nut) checklist. **Table S2:** Diet group categorisation and definitions. **Table S3:** Covariates at recruitment and their derivation. **Table S4:** Further dietary characteristics of UKWCS participants by diet group at recruitment. **Table S5:** Characteristics of UKWCS participants at recruitment that were included or excluded from adjusted analyses. **Table S6:** Risk of hip fracture in occasional meat-eaters, pescatarians, and vegetarians compared to regular meat-eaters stratified by potential effect modifiers in the UKWCS. **Table S7:** Risk of hip fracture by diet group with varying restrictions in the UKWCS. **Supplementary methods**.

## Data Availability

The data access policy for the UK Women’s Cohort Study is available via the study website (The UK Women’s Cohort Study (UKWCS) : University of Leeds).

## Abbreviations

BMD: Bone mineral density
BMI: Body mass index
UKWCS: United Kingdom’s Women’s Cohort Study
EPIC: European Prospective Investigation into Cancer and Nutrition
AHS-2: Adventist Health Study-2
ICD: International Classification of Diseases
IGF-1: Insulin-like growth factor-1
MUFA: Monounsaturated fatty acids
PUFA: Polyunsaturated fatty acids

## References

[1] Dhanwal DK, Dennison EM, Harvey NC, Cooper C (2011). Epidemiology of hip fracture: worldwide geographic variation. Indian J Orthop.

[2] Veronese N, Maggi S (2018). Epidemiology and social costs of hip fracture. Injury.

[3] Jennison T, Brinsden M (2019). Fracture admission trends in England over a ten-year period. Ann R Coll Surg Engl.

[4] Peraza-Delgado A, Sánchez-Gómez MB, Gómez-Salgado J, Romero-Martín M, Novo-Muñoz M, Duarte-Clíments G (2020). Non-pharmacological interventions towards preventing the triad osteoporosis-falls risk-hip fracture, in population older than 65. Scoping Review. J Clin Med.

[5] Williamson S, Landeiro F, McConnell T, Fulford-Smith L, Javaid MK, Judge A (2017). Costs of fragility hip fractures globally: a systematic review and meta-regression analysis. Osteoporos Int.

[6] Tucker KL (2014). Vegetarian diets and bone status. Am J Clin Nutr.

[7] Iguacel I, Miguel-Berges ML, Gómez-Bruton A, Moreno LA, Julián C (2019). Veganism, vegetarianism, bone mineral density, and fracture risk: a systematic review and meta-analysis. Nutr Rev.

[8] Tong TYN, Appleby PN, Armstrong MEG, Fensom GK, Knuppel A, Papier K (2020). Vegetarian and vegan diets and risks of total and site-specific fractures: results from the prospective EPIC-Oxford study. BMC Med.

[9] Thorpe DL, Beeson WL, Knutsen R, Fraser GE, Knutsen SF (2021). Dietary patterns and hip fracture in the Adventist Health Study 2: combined vitamin D and calcium supplementation mitigate increased hip fracture risk among vegans. Am J Clin Nutr.

[10] Le LT, Sabaté J (2014). Beyond meatless, the health effects of vegan diets: findings from the Adventist cohorts. Nutrients.

[11] Foods Standards Agency (2012). National diet and nutrition survey: headline results from Years 1 and 2 (combined) of the Rolling Programme (2009/2009 - 2009/10).

[12] Benson A, Irdam D, Bulceag I, Barber T, Draper A (2019). Food and you survey: wave 5 secondary analysis: the current food landscape across England, Wales, and Northern Ireland.

[13] Shridhar K, Dhillon PK, Bowen L, Kinra S, Bharathi AV, Prabhakaran D (2014). Nutritional profile of Indian vegetarian diets--the Indian Migration Study (IMS). Nutr J.

[14] Lee Y, Park K (2017). Adherence to a vegetarian diet and diabetes risk: a systematic review and meta-analysis of Observational Studies. Nutrients.

[15] Dinu M, Abbate R, Gensini GF, Casini A, Sofi F (2017). Vegetarian, vegan diets and multiple health outcomes: a systematic review with meta-analysis of observational studies. Crit Rev Food Sci Nutr.

[16] Rosi A, Mena P, Pellegrini N, Turroni S, Neviani E, Ferrocino I (2017). Environmental impact of omnivorous, ovo-lacto-vegetarian, and vegan diet. Sci Rep.

[17] Fresán U, Sabaté J (2019). Vegetarian diets: planetary health and its alignment with human health. Adv Nutr.

[18] Luo S, Li Y, Luo H, Yin X, Lin du R, Zhao K (2016). Increased intake of vegetables, but not fruits, may be associated with reduced risk of hip fracture: a meta-analysis. Sci Rep.

[19] Brondani JE, Comim FV, Flores LM, Martini LA, Premaor MO (2019). Fruit and vegetable intake and bones: a systematic review and meta-analysis. PLoS One.

[20] Webster J, Rycroft CE, Greenwood DC, Cade JE (2021). Dietary risk factors for hip fracture in adults: an umbrella review of meta-analyses of prospective cohort studies. PLoS One.

[21] Lousuebsakul-Matthews V, Thorpe DL, Knutsen R, Beeson WL, Fraser GE, Knutsen SF (2014). Legumes and meat analogues consumption are associated with hip fracture risk independently of meat intake among Caucasian men and women: the Adventist Health Study-2. Public Health Nutr.

[22] Davey GK, Spencer EA, Appleby PN, Allen NE, Knox KH, Key TJ (2003). EPIC-Oxford: lifestyle characteristics and nutrient intakes in a cohort of 33 883 meat-eaters and 31 546 non meat-eaters in the UK. Public Health Nutr.

[23] Bradbury KE, Tong TYN, Key TJ (2017). Dietary intake of high-protein foods and other major foods in meat-eaters, poultry-eaters, fish-eaters, vegetarians, and vegans in UK Biobank. Nutrients.

[24] Armstrong ME, Cairns BJ, Banks E, Green J, Reeves GK, Beral V (2012). Different effects of age, adiposity and physical activity on the risk of ankle, wrist and hip fractures in postmenopausal women. Bone.

[25] Ho-Pham LT, Nguyen ND, Nguyen TV (2009). Effect of vegetarian diets on bone mineral density: a Bayesian meta-analysis. Am J Clin Nutr.

[26] Tong TY, Key TJ, Sobiecki JG, Bradbury KE (2018). Anthropometric and physiologic characteristics in white and British Indian vegetarians and nonvegetarians in the UK Biobank. Am J Clin Nutr.

[27] Cade JE, Burley VJ, Alwan NA, Hutchinson J, Hancock N, Morris MA (2017). Cohort profile: the UK Women’s Cohort Study (UKWCS). Int J Epidemiol.

[28] Lachat C, Hawwash D, Ocké MC, Berg C, Forsum E, Hörnell A (2016). Strengthening the Reporting of Observational Studies in Epidemiology-Nutritional Epidemiology (STROBE-nut): an extension of the STROBE Statement. PLoS Med.

[29] Rada-Fernandez de Jauregui D, Evans CEL, Jones P, Greenwood DC, Hancock N, Cade JE (2018). Common dietary patterns and risk of cancers of the colon and rectum: analysis from the United Kingdom Women’s Cohort Study (UKWCS). Int J Cancer.

[30] Korn EL, Graubard BI, Midthune D (1997). Time-to-event analysis of longitudinal follow-up of a survey: choice of the time-scale. Am J Epidemiol.

[31] Tennant PWG, Murray EJ, Arnold KF, Berrie L, Fox MP, Gadd SC (2021). Use of directed acyclic graphs (DAGs) to identify confounders in applied health research: review and recommendations. Int J Epidemiol.

[32] Benetos IS, Babis GC, Zoubos AB, Benetou V, Soucacos PN (2007). Factors affecting the risk of hip fractures. Injury.

[33] Malmir H, Saneei P, Larijani B, Esmaillzadeh A (2018). Adherence to Mediterranean diet in relation to bone mineral density and risk of fracture: a systematic review and meta-analysis of observational studies. Eur J Nutr.

[34] Panahande B, Sadeghi A, Parohan M (2019). Alternative healthy eating index and risk of hip fracture: a systematic review and dose-response meta-analysis. J Hum Nutr Diet.

[35] Fabiani R, Naldini G, Chiavarini M (2019). Dietary patterns in relation to low bone mineral density and fracture risk: a systematic review and meta-analysis. Adv Nutr (Bethesda).

[36] Barnard ND, Levin SM, Yokoyama Y (2015). A systematic review and meta-analysis of changes in body weight in clinical trials of vegetarian diets. J Acad Nutr Diet.

[37] Kim SH, Yi SW, Yi JJ, Kim YM, Won YJ (2018). Association between body mass index and the risk of hip fracture by sex and age: a prospective cohort study. J Bone Miner Res.

[38] Gonnelli S, Caffarelli C, Nuti R (2014). Obesity and fracture risk. Clin Cases Miner Bone Metab.

[39] Hayes WC, Myers ER, Morris JN, Gerhart TN, Yett HS, Lipsitz LA (1993). Impact near the hip dominates fracture risk in elderly nursing home residents who fall. Calcif Tissue Int.

[40] Kim KH, Lee JH, Lim EJ (2021). Weak psoas and spine extensors potentially predispose to hip fracture. Hip Int.

[41] Wu AM, Sun XL, Lv QB, Zhou Y, Xia DD, Xu HZ (2015). The relationship between dietary protein consumption and risk of fracture: a subgroup and dose-response meta-analysis of prospective cohort studies. Sci Rep.

[42] Bailey RL, van Wijngaarden JP (2015). The role of B-vitamins in bone health and disease in older adults. Curr.

[43] British Nutrition Foundation (2021). Nutrition requirements.

[44] Yao P, Bennett D, Mafham M, Lin X, Chen Z, Armitage J (2019). Vitamin D and calcium for the prevention of fracture: a systematic review and meta-analysis. JAMA Netw Open.

[45] Allen NE, Appleby PN, Davey GK, Kaaks R, Rinaldi S, Key TJ (2002). The associations of diet with serum insulin-like growth factor I and its main binding proteins in 292 women meat-eaters, vegetarians, and vegans. Cancer Epidemiol Biomark Prev.

[46] Yuan S, Wan ZH, Cheng SL, Michaëlsson K, Larsson SC (2021). Insulin-like growth factor-1, bone mineral density, and fracture: a Mendelian randomization study. J Clin Endocrinol Metab.

[47] Swift CG (2011). Prevention and management of hip fracture in older patients. Practitioner..

[48] Robinson CM, Court-Brown CM, McQueen MM, Christie J (1995). Hip fractures in adults younger than 50 years of age. Epidemiology and results. Clin Orthop Relat Res.

[49] Curtain F, Grafenauer S (2019). Plant-based meat substitutes in the flexitarian age: an audit of products on supermarket shelves. Nutrients.

[50] Barrett-Connor E, Siris ES, Wehren LE, Miller PD, Abbott TA, Berger ML (2005). Osteoporosis and fracture risk in women of different ethnic groups. J Bone Miner Res.

