# Supplementary material for: Risk of hip fracture in meat-eaters, pescatarians, and vegetarians: results from the UK Women’s Cohort Study
Source: BMC Med. 2022 Aug 11;20:275. doi: 10.1186/s12916-022-02468-0 (PMC9367078; doi:10.1186/s12916-022-02468-0)
Supplement: Supplementary file 1 — Additional file 1: Figure S1. Flow chart of UKWCS participants. Figure S2. Directed Acyclic Graph showing the relationship between diet group, hip fracture incidence, and related factors. Table S1: Strengthening the reporting of observational studies in nutritional epidemiology (STROBE-Nut) checklist. Table S2: Diet group categorisation and definitions. Table S3: Covariates at recruitment and their derivation. Table S4: Further dietary characteristics of UKWCS participants by diet group at recruitment. Table S5: Characteristics of UKWCS participants at recruitment that were included or excluded from adjusted analyses. Table S6: Risk of hip fracture in occasional meat-eaters, pescatarians, and vegetarians compared to regular meat-eaters stratified by potential effect modifiers in the UKWCS. Table S7: Risk of hip fracture by diet group with varying restrictions in the UKWCS. Supplementary methods. [file 12916_2022_2468_MOESM1_ESM.docx]

**Additional file 1**

Contents

**Supplementary figures**2-3

Fig S1: Flow chart of UKWCS participants2

Fig S2: Directed Acyclic Graph showing the relationship between diet group, hip fracture incidence, and related factors3

**Supplementary tables**4-18

Table S1: Strengthening the reporting of observational studies in nutritional epidemiology (STROBE-Nut) checklist4-6

Table S2: Diet group categorisation and definitions7

Table S3: Covariates at recruitment and their derivation8-9

Table S4: Further dietary characteristics of UKWCS participants by diet group at recruitment10-11

Table S5: Characteristics of UKWCS participants at recruitment that were included or excluded from adjusted analyses12-13

Table S6: Risk of hip fracture in occasional meat-eaters, pescatarians, and vegetarians compared to regular meat-eaters stratified by potential effect modifiers in the UKWCS14-15

Table S7: Risk of hip fracture by diet group with varying restrictions in the UKWCS16-17

**Supplementary methods**18

# **Supplementary Figures**

**Fig S1: Flow chart of UKWCS participants.**

UK Women’s Cohort Study participants recruited between 1995 – 1998 (n = 35,372)

Participants potentially eligible (n = 30,244)

- 13,984 regular meat-eaters
- 8000 occasional meat-eaters
- 3867 pescatarians
- 4393 vegetarians

**Exclusion criteria (n total = 5128):**

- Not resident in England (n = 3821)
- Had a hip fracture before or on the data of recruitment (n = 2)
- Outlier nutrient or covariate data (n = 941)
- Missing age data (n = 364)

Participants in unadjusted and multivariable-adjusted analyses with complete covariate data (n = 26,318)

- 12,221 regular meat-eaters
- 6902 occasional meat-eaters
- 3377 pescatarians
- 3818 vegetarians

**Excluded due to missing covariate data (n total = 3926):**

- Weight (n = 596)
- Height (n = 649)
- Ethnicity (n = 811)
- Physical activity (n = 1561)
- Marital status (n = 460)
- Socio-economic status (n = 331)
- Menopausal status (n = 309)

**Fig S1: Flow chart of UKWCS participants.**


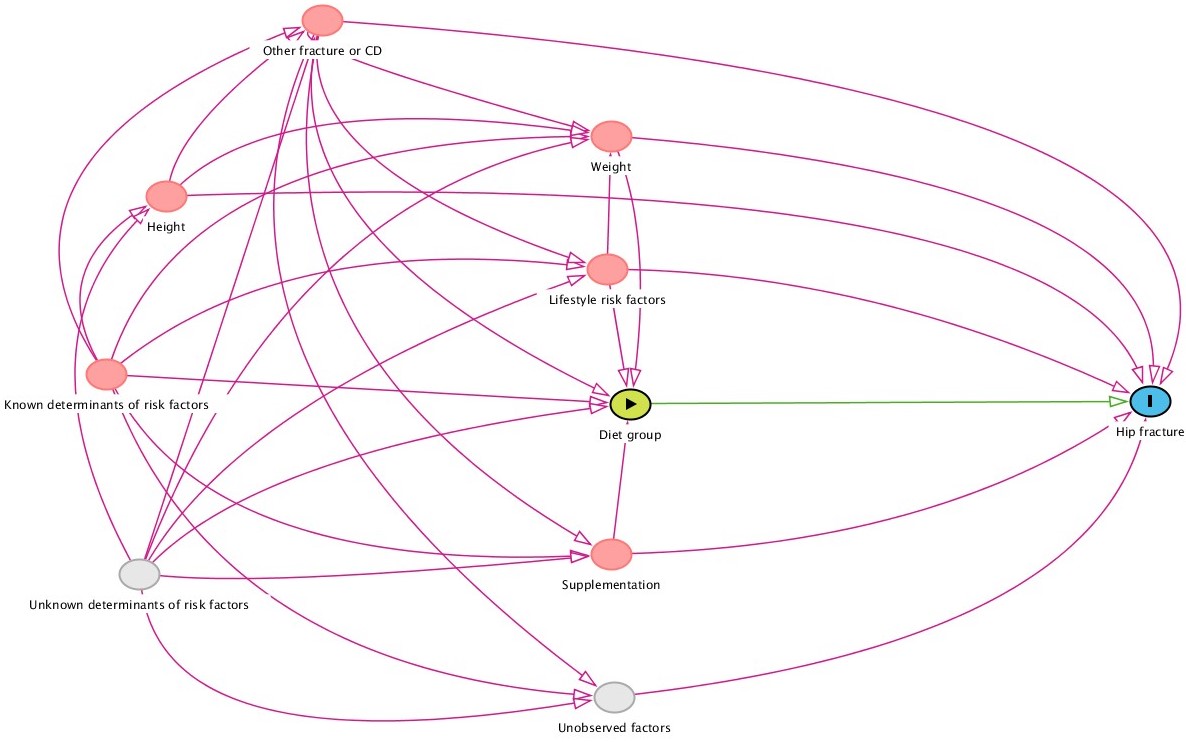


The exposure (diet group) is depicted by the green oval and the outcome (hip fracture incidence) is depicted by the blue node with a black vertical line. Variables represented as pink nodes are ancestors of the exposure and outcome, whilst variables represented as grey nodes are unknown or unmeasured. The green line represents the causal link of interest, whilst pink lines are biasing paths. Known determinants of risk factors include age, ethnicity, education, socioeconomic status, marital status, menopausal status, and number of children. Lifestyle risk factors include physical activity, smoking, and alcohol intake. Supplementation refers to use of any nutritional supplements. CD: chronic disease, defined as prevalence of cardiovascular disease, cancer, diabetes, or osteoporosis.

### Fig S2: Directed Acyclic Graph showing the relationship between diet group, hip fracture incidence, and related factors.

# **Supplementary Tables**

**Table S1: Strengthening the reporting of observational studies in nutritional epidemiology (STROBE-Nut) checklist.**

| **Section/topic** | **Item number** | **Recommendation** | **Page (line number)** |
| --- | --- | --- | --- |
| Title and abstract | 1 | Indicate the study’s design with a commonly used term in the title or the abstract | 1-2 (2-3, 16) |
|  |  | Provide in the abstract an informative and balanced summary of what was done and what was found | 2-3 (12-35) |
| Introduction |  |  |  |
| Background/rationale | 2 | Explain the scientific background and rationale for the investigation being reported | 4-5 (39-71) |
| Objectives | 3 | State specific objectives, including any prespecified hypotheses | 5 (72-76) |
| Methods |  |  |  |
| Study design | 4 | Present key elements of study design early in the manuscript | 5-6 (81-88) |
| Setting | 5 | Describe the setting, locations, and relevant dates, including periods of recruitment, exposure, follow-up, and data collection | 5-6 (81-88) |
| Participants | 6 | Cohort study - give the eligibility criteria, and the sources and methods of selection of participants; describe methods of follow-up | 6 (91-98), Additional file 1: Fig S1 |
|  |  | Cohort study - for matched studies, give matching criteria and number of exposed and unexposed Case-control study - for matched studies, give matching criteria and the number of controls per case | N/A |
| Variables | 7 | Clearly define all outcomes, exposures, predictors, potential confounders, and effect modifiers; give diagnostic criteria, if applicable | 6-7 (99-110), 7 (111-117), 7-8 (129-138); 8 (140-149); Additional file 1: Tables S2 and S3 |
| Data sources/measurement | 8* | For each variable of interest, give sources of data and details of methods of assessment (measurement); describe comparability of assessment methods if there is more than one group | 6-7 (99-110), 7 (111-117), 7-8 (129-138); 8 (140-149); Additional file 1: Tables S2 and S3 |
| Bias | 9 | Describe any efforts to address potential sources of bias | 7-8 (125-139), 8-9 (155-165) |
| Study size | 10 | Explain how the study size was arrived at | 6 (91-95); 9 (166-171); Additional file 1: Fig S1 |
| Quantitative variables | 11 | Explain how quantitative variables were handled in the analyses; if applicable, describe which groupings were chosen and why | 8 (140-149), Additional file 1: Supplementary methods, Table S2, and Table S3 |
| Statistical methods | 12 | Describe all statistical methods, including those used to control for confounding | 7-9 (118-165); Additional file 1: Supplementary methods and Fig S2 |
|  |  | Describe any methods used to examine subgroups and interactions | 8 (140-149) |
|  |  | Explain how missing data were addressed | 9 (163-165) |
|  |  | Cohort study - if applicable, explain how loss to follow-up was addressed | N/A |
|  |  | Describe any sensitivity analyses | 8-9 (154-164) |
| Results |  |  |  |
| Participants | 13* | Report numbers of individuals at each stage of study - e.g., numbers potentially eligible, examined for eligibility, confirmed eligible, included in the study, completing follow-up, and analyzed | 9 (166-171), Additional file 1: Fig S1 |
|  |  | Give reasons for nonparticipation at each stage Consider use of a flow diagram | 9 (166-171), Additional file 1: Fig S1 |
| Descriptive data | 14* | Give characteristics of study participants (e.g., demographic, clinical, social) and information on exposures and potential confounders | 9-10 (172-187); Table 1 |
|  |  | Indicate number of participants with missing data for each variable of interest Cohort study - summarize follow-up time (e.g., average and total amount) | 9 (168-175); Additional file 1: Fig S1 |
| Outcome data | 15* | Cohort study - report numbers of outcome events or summary measures over time | 9 (173-175) |
| Main results | 16 | Give unadjusted estimates and, if applicable, confounder-adjusted estimates and their precision (e.g., 95% confidence interval); make clear which confounders were adjusted for and why they were included Report category boundaries when continuous variables were categorized | 10 (189-195); Fig 1; Additional file 1: Table S3 |
|  |  | If relevant, consider translating estimates of relative risk into absolute risk for a meaningful time period | N/A |
| Other analyses | 17 | Report other analyses done - e.g., analyses of subgroups and interactions, and sensitivity analyses | 12-14 (196-217); Tables 2-3, Additional file 1: Tables S6 and S7. |
| Discussion |  |  |  |
| Key results | 18 | Summarize key results with reference to study objectives | 16 (218-224) |
| Limitations | 19 | Discuss limitations of the study, taking into account sources of potential bias or imprecision; discuss both direction and magnitude of any potential bias | 19-20 (293-328) |
| Interpretation | 20 | Give a cautious overall interpretation of results considering objectives, limitations, multiplicity of analyses, results from similar studies, and other relevant evidence | 17-19 (258-292) |
| Generalizability | 21 | Discuss the generalizability (external validity) of the study results | 20 (322-328) |
| Other information |  |  |  |
| Funding | 22 | Give the source of funding and the role of the funders for the present study and, if applicable, for the original study on which the present article is based | 21 (354-356) |

**Table S2: Diet group categorisation and definitions.**

| **Diet group** | **Definition** |
| --- | --- |
| Regular meat-eater | Total meat intake ≥ 5 servings/week |
| Occasional meat-eater | Total meat intake < 5 servings/week & ≥ 1 serving/month |
| Pescatarian | Total meat intake < 1 serving/month & total fish intake ≥ 1 serving/month |
| Vegetarian | Total meat and fish intakes < 1 serving/month, intake of dairy products or eggs ≥ 1 serving/month |
| Vegan | Total meat, total fish, dairy products, and eggs intake < 1 serving/month |

### Table S3: Covariates at recruitment and their derivation.

| **Covariate** | **How the variable was derived** |
| --- | --- |
| **Socio-demographic variables** | |
| Age | Calculated as year differences between date of birth and date of recruitment and was considered a continuous variable in adjustment sets. |
| Ethnicity | Participants were asked to select which ethnic group they belong to of ‘white, ‘Bangladeshi’, ‘Indian’, ‘Chinese’, ‘Pakistani’, ‘Black-Caribbean’, ‘Black – other’, ‘other’. We regrouped ethnicity into ‘White’, ‘Asian’, ‘Black’, and ‘Other’. |
| Socio-economic status | Participants were asked about their occupation. Options were ‘never had paid job’, ‘managers and administrators’, ‘professional’, ‘technical and associate professional’, ‘clerical and secretarial’, ‘craft and skilled’, ‘personal and protective’, ‘sales’, ‘plant and machine operatives’, or ‘other’. We condensed these options into ‘routine/manual’, ‘intermediate’, or ‘managerial/professional’. |
| Education | Participants were asked what their highest educational qualification was. Options were ‘no qualifications’, ‘O level’, ‘A level’, ‘degree’, or ‘missing’. |
| Marriage | Participants were asked ‘what is your marital status?’ with options of ‘married or living as married’, ‘divorced’, ‘widowed’, ‘single’, or ‘separated’. We combined ‘divorced’ and ‘separated’ together, and ‘widowed’ and ‘single’ together. |
| **Lifestyle and other variables** | |
| Physical activity | Participants were asked how long they perform exercises that makes them sweat per week (in hours and minutes per week). This was computed as hours per day. |
| Smoking | Participants were asked to describe their smoking habit as ‘smoke daily’, ‘smoke occasionally’, ‘ex-smoker’, or ‘never’. We combined daily and occasional smokers into ‘smokers’, and kept ‘ex-smoker’ and ‘never smoked’ the same. |
| Alcohol | Participants were asked how often they drink alcohol. Options were ‘> 1/week’, ‘1/week’, ‘< 1/week’, or ‘never’. This was regrouped as ‘≥ 1/week’, ‘< 1/week’, or ‘never’. |
| Body weight | Self-reported continuous variable |
| Height | Self-reported continuous variable |
| Body mass index | Calculated as self-reported weight divided by the square of self-reported height, considered as a continuous variable |
| Number of children | Self-reported continuous variable |
| Menopausal status | Categorised participants as pre-menopausal or post-menopausal. Criteria for postmenopausal was: age > 55 years, both ovaries removed, currently on hormone replacement therapy, or no periods in the last 12 months. |
| Hormone replacement therapy use | Participants were asked ‘have you ever used hormone replacement therapy?’ and ‘are you using HRT now?’ – based on these yes or no answers, we categorised hormone replacement therapy use as ‘current’, ‘ex-user’, and ‘never’. |

**Table S4: Further dietary characteristics of the UKWCS by diet group at recruitment.**

| **Characteristics** | **Total** | **Regular meat-eater** | **Occasional meat-eater** | **Pescatarian** | **Vegetarian** |
| --- | --- | --- | --- | --- | --- |
| Participants | 30244 | 13984 | 8000 | 3867 | 4393 |
| **Foods and beverages intake** |  |  |  |  |  |
| Total fruit and vegetable (g/day) | 647.7 (301.4) | 626.1 (290.7) | 630.8 (306.8) | 707.4 (309.2) | 694.9 (305.9) |
| Fruit (g/day) | 376.6 (228.4) | 358.3 (220.0) | 376.4 (232.4) | 411.8 (236.1) | 403.9 (234.0) |
| Vegetable (g/day) | 271.1 (140.2) | 267.7 (135.3) | 254.4 (141.2) | 295.6 (145.2) | 291.0 (144.0) |
| Total meat (g/day) | 86.1 (81.2) | 155.5 (63.2) | 53.7 (29.1) | 0.1 (0.5) | 0.0 (0.2) |
| Red meat (g/day) | 39.4 (47.1) | 73.3 (48.2) | 20.9 (19.7) | 0.0 (0.1) | 0.0 (0.1) |
| Poultry (g/day) | 16.6 (20.1) | 27.9 (22.1) | 14.0 (12.5) | 0.1 (0.5) | 0.0 (0.1) |
| Processed meat (g/day) | 28.2 (30.9) | 51.0 (30.0) | 17.4 (14.1) | 0.0 (0.2) | 0.0 (0.1) |
| Offal (g/day) | 1.9 (3.6) | 3.4 (4.5) | 1.3 (2.5) | 0.0 (0.1) | 0.0 (0.0) |
| Fish (g/day) | 33.7 (29.7) | 40.0 (26.0) | 37.9 (29.7) | 40.1 (33.9) | 0.1 (0.5) |
| Oily fish (g/day) | 8.9 (12.0) | 9.8 (10.9) | 10.6 (13.1) | 12.2 (15.3) | 0.1 (0.3) |
| Non-oily fish (g/day) | 24.7 (23.1) | 30.2 (20.9) | 27.2 (23.0) | 27.9 (25.9) | 0.1 (0.5) |
| Total dairy (g/day) | 411.2 (215.4) | 438.4 (202.8) | 402.5 (213.7) | 391.1 (225.7) | 358.5 (234.6) |
| Milk (g/day) | 302.8 (191.5) | 329.9 (182.2) | 299.0 (190.8) | 273.8 (196.5) | 248.6 (201.5) |
| Yoghurt (g/day) | 59.4 (68.9) | 58.8 (65.9) | 60.4 (70.7) | 64.0 (72.7) | 55.4 (71.1) |
| Cheese (g/day) | 27.3 (27.5) | 23.5 (23.3) | 24.7 (26.1) | 35.2 (29.6) | 37.3 (35.2) |
| Cream (g/day) | 1.7 (3.4) | 2.2 (3.9) | 1.4 (3.2) | 1.3 (2.4) | 1.2 (2.6) |
| Dairy desserts (g/day) | 20.1 (26.1) | 23.9 (27.8) | 17.0 (23.6) | 16.9 (24.2) | 16.1 (24.4) |
| Eggs (number/day) | 0.3 (0.2) | 0.3 (0.3) | 0.2 (0.2) | 0.3 (0.2) | 0.2 (0.3) |
| Tea (cups/day) | 3.0 (2.0) | 3.0 (2.0) | 2.9 (2.0) | 3.1 (2.1) | 3.0 (2.1) |
| Caffeinated coffee (cups/day) | 1.5 (1.7) | 1.6 (1.7) | 1.4 (1.6) | 1.3 (1.6) | 1.3 (1.6) |
| Decaffeinated coffee (cups/day) | 0.5 (1.1) | 0.5 (1.1) | 0.5 (1.1) | 0.5 (1.1) | 0.5 (1.1) |
| Tea and coffee (cups/day) | 5.0 (2.2) | 5.2 (2.1) | 4.8 (2.2) | 4.9 (2.2) | 4.8 (2.3) |
| **Nutrients** |  |  |  |  |  |
| Protein (g/kg BW/day) | 1.37 (0.45) | 1.53 (0.45) | 1.23 (0.40) | 1.29 (0.42) | 1.21 (0.41) |
| Carbohydrate (g/day) | 303.6 (94.2) | 309.6 (92.3) | 283.0 (92.1) | 313.3 (95.2) | 313.5 (97.0) |
| Carbohydrate (% energy) | 53.0 (6.7) | 50.6 (6.0) | 54.7 (6.6) | 54.8 (6.5) | 55.9 (6.8) |
| Fibre intake (g/day) | 24.7 (9.1) | 23.7 (8.5) | 23.5 (9.2) | 27.6 (9.5) | 27.6 (9.5) |
| Fibre (% energy) | 2.2 (0.6) | 2.0 (0.5) | 2.3 (0.6) | 2.4 (0.6) | 2.5 (0.6) |
| Fat (g/day) | 83.0 (30.5) | 90.4 (29.8) | 71.4 (27.2) | 82.4 (30.9) | 81.3 (31.2) |
| Fat (% energy) | 32.3 (5.6) | 33.2 (5.0) | 30.8 (5.8) | 32.0 (6.0) | 32.2 (6.2) |
| Saturated fat (g/day) | 28.8 (12.8) | 32.5 (12.7) | 24.5 (11.1) | 27.1 (12.5) | 26.4 (12.6) |
| Saturated fat (% energy) | 11.2 (3.1) | 11.9 (2.9) | 10.6 (3.2) | 10.5 (3.1) | 10.4 (3.4) |
| MUFA (g/day) | 27.2 (10.5) | 30.1 (10.1) | 23.3 (9.4) | 26.7 (10.7) | 26.0 (10.9) |
| MUFA (% energy) | 10.6 (2.2) | 11.0 (1.9) | 10.1 (2.3) | 10.4 (2.4) | 10.3 (2.5) |
| PUFA (g/day) | 15.9 (6.4) | 16.2 (5.9) | 14.0 (6.1) | 17.3 (6.9) | 17.3 (7.4) |
| PUFA (% energy) | 6.2 (1.6) | 6.0 (1.4) | 6.0 (1.7) | 6.8 (1.8) | 6.9 (2.0) |
| Vitamin c (mg/day) | 165.1 (71.0) | 166.4 (69.9) | 157.2 (70.9) | 172.5 (73.1) | 169.0 (71.4) |
| Zinc (mg/day) | 11.2 (3.6) | 12.7 (3.5) | 9.8 (2.9) | 10.2 (3.2) | 10.0 (3.2) |
| Phosphorus (mg/day) | 1672 (623) | 1753 (620) | 1540 (621) | 1697 (597) | 1629 (616) |
| Magnesium (mg/day) | 399 (144) | 390 (135) | 375 (144) | 438 (146) | 439 (154) |
| Selenium (µg/day) | 57.7 (23.5) | 66.3 (23.8) | 53.0 (21.4) | 55.3 (20.8) | 41.2 (15.4) |

BW: body weight.

**Table S5: Characteristics of UKWCS participants at recruitment that were included or excluded from adjusted analyses.**

| **Characteristics, mean (SD) or n (%)** | **30,244 potentially eligible participants** | | | | **3,926 participants excluded from adjusted analyses** | | | |
| --- | --- | --- | --- | --- | --- | --- | --- | --- |
| Diet group | **Regular meat-eater** | **Occasional meat-eater** | **Pescatarian** | **Vegetarian** | **Regular meat-eater** | **Occasional meat-eater** | **Pescatarian** | **Vegetarian** |
| Participants (%) | 13984 (46.2) | 8000 (26.5) | 3867 (12.8) | 4393 (14.5) | 1763 (44.9) | 1098 (30.0) | 490 (12.5) | 575 (14.6) |
| Cases (%) | 471 (3.4) | 296 (3.7) | 98 (2.5) | 128 (2.9) | 77 (4.4) | 49 (4.5) | 18 (3.7) | 27 (4.7) |
| **Socio-demographics** |  |  |  |  |  |  |  |  |
| Age, years (SD) | 53.6 (9.3) | 53.5 (9.5) | 49.8 (8.6) | 48.4 (8.3) | 55.6 (9.9) | 55.2 (9.9) | 50.6 (9.0) | 49.4 (9.1) |
| Degree-level education (%) | 2575 (18.4) | 2022 (25.3) | 1327 (34.3) | 1460 (33.2) | 269 (15.3) | 242 (22.0) | 184 (37.6) | 187 (32.5) |
| SES |  |  |  |  |  |  |  |  |
| Professional or managerial (%) | 9529 (68.1) | 5802 (72.5) | 2905 (75.1) | 3208 (73.0) | 1011 (57.3) | 682 (62.1) | 329 (67.1) | 365 (63.5) |
| Intermediate (%) | 1342 (9.6) | 840 (10.5) | 321 (8.3) | 421 (9.6) | 225 (12.8) | 146 (13.3) | 51 (10.4) | 62 (10.8) |
| Routine or manual (%) | 2978 (21.3) | 1265 (15.8) | 607 (15.7) | 695 (15.8) | 392 (22.2) | 177 (16.1) | 76 (15.5) | 79 (13.7) |
| Married (%) | 11183 (80.0) | 5566 (69.6) | 2663 (68.9) | 3002 (68.3) | 1080 (61.3) | 559 (50.9) | 231 (47.%) | 276 (48.0) |
| White ethnicity (%) | 13577 (97.1) | 7652 (95.7) | 3690 (95.4) | 4126 (93.9) | 1438 (81.6) | 832 (75.8) | 359 (73.3) | 424 (73.7) |
| **Lifestyle** |  |  |  |  |  |  |  |  |
| Exercise (hours/day) | 0.2 (0.5) | 0.2 (0.5) | 0.3 (0.5) | 0.3 (0.5) | 0.2 (0.4) | 0.2 (0.5) | 0.2 (0.4) | 0.3 (0.4) |
| Smoking status |  |  |  |  |  |  |  |  |
| Current | 1998 (14.3) | 1121 (14.0) | 516 (13.3) | 555 (12.6) | 320 (18.2) | 200 (18.2) | 68 (13.9) | 89 (15.5) |
| Former | 4037 (28.9) | 2409 (30.1) | 1328 (34.3) | 1366 (31.1) | 518 (29.4) | 331 (30.1) | 167 (34.1) | 177 (30.8) |
| Never | 7949 (56.8) | 4470 (55.9) | 2023 (52.3) | 2472 (56.3) | 925 (52.5) | 567 (51.6) | 255 (52.0) | 309 (53.7) |
| Alcohol consumption |  |  |  |  |  |  |  |  |
| > 1/week (%) | 7646 (54.7) | 4020 (50.2) | 2051 (53.0) | 1944 (44.3) | 848 (48.1) | 472 (43.0) | 221 (45.1) | 202 (35.1) |
| ≤ 1/week (%) | 4924 (35.2) | 2893 (36.2) | 1317 (34.1) | 1605 (36.5) | 648 (36.8) | 422 (38.4) | 172 (35.1) | 207 (36.0) |
| Never (%) | 1414 (10.1) | 1087 (13.6) | 499 (12.9) | 844 (19.2) | 267 (15.1) | 204 (18.6) | 97 (19.8) | 166 (28.9) |
| Nutritional supplementation (%) | 6701 (47.9) | 4475 (55.9) | 2347 (60.7) | 2470 (56.2) | 799 (45.3) | 594 (54.1) | 277 (56.5) | 314 (54.6) |
| **Anthropometrics** |  |  |  |  |  |  |  |  |
| BMI, kg/m^2^ | 25.3 (4.5) | 24.1 (3.9) | 23.3 (3.5) | 23.3 (3.9) | 25.8 (4.8) | 24.2 (4.0) | 23.6 (3.6) | 23.2 (4.0) |
| Height (m) | 1.6 (0.1) | 1.6 (0.1) | 1.6 (0.1) | 1.6 (0.1) | 1.6 (0.1) | 1.6 (0.1) | 1.6 (0.1) | 1.6 (0.1) |
| **Diet** |  |  |  |  |  |  |  |  |
| Energy intake (kcal/day) | 2439 (643) | 2065 (613) | 2290 (659) | 2250 (663) | 2397 (657.4) | 2043 (652.3) | 2262 (679.4) | 2189 (693.6) |
| Protein intake (% energy) | 100.8 (25.0) | 77.3 (21.5) | 79.3 (23.2) | 73.6 (22.5) | 16.9 (2.6) | 15.2 (2.3) | 13.8 (2.3) | 13.1 (1.9) |
| Protein intake (g/day) | 100.9 (24.8) | 77.5 (21.3) | 79.6 (23.1) | 74.0 (22.3) | 99.6 (26.0) | 76.3 (22.7) | 77.5 (24.0) | 71.2 (23.4) |
| Protein intake (g/kg-BW/day) | 1.5 (0.4) | 1.2 (0.4) | 1.3 (0.4) | 1.2 (0.4) | 1.5 (0.5) | 1.2 (0.4) | 1.2 (0.4) | 1.2 (0.4) |
| Calcium intake (mg/day) | 1160 (346) | 1059 (360) | 1177.4 (398) | 1131 (400) | 1142 (357.8) | 1053 (385.0) | 1143 (413.9) | 1089 (407.1) |
| Vitamin D intake (µg/day) | 3.6 (1.6) | 2.9 (1.6) | 3.1 (1.8) | 1.9 (1.1) | 3.6 (1.7) | 2.9 (1.6) | 2.9 (1.7) | 1.8 (1.2) |
| Vitamin B12 intake (µg/day) | 7.5 (2.9) | 5.1 (2.2) | 4.2 (2.0) | 2.5 (1.2) | 7.4 (3.1) | 5.0 (2.1) | 4.0 (2.0) | 2.4 (1.2) |
| **Other** |  |  |  |  |  |  |  |  |
| Postmenopausal (%) | 5154 (36.9) | 3103 (38.8) | 2062 (53.3) | 2667 (60.7) | 1194 (67.7) | 689 (62.8) | 218 (44.5) | 237 (41.2) |
| Premenopausal (%) | 8715 (62.3) | 4803 (60.0) | 1749 (45.2) | 1682 (38.3) | 454 (25.8) | 315 (28.7) | 216 (44.1) | 294 (51.1) |
| ≥ 1 children (%) | 11607 (83.0) | 6087 (76.1) | 2785 (72.0) | 3033 (69.0) | 1344 (76.2) | 763 (69.5) | 317 (64.7) | 365 (63.5) |
| Chronic disease prevalence (%) | 1459 (10.4) | 773 (9.7) | 300 (7.8) | 263 (6.0) | 209 (11.9) | 109 (9.9) | 48 (9.8) | 41 (7.1) |

Chronic disease prevalence includes stroke, diabetes or cancer at baseline. SD: standard deviation; SES: social economic status; BMI: body mass index.

**Table S6: Risk of hip fracture in occasional meat-eaters, pescatarians, and vegetarians compared to regular meat-eaters stratified by potential effect modifiers in the UKWCS.**

| **Stratifying variable** | **n cases, adjusted HR (95% CI)** | | | | | | ***p* interaction** |
| --- | --- | --- | --- | --- | --- | --- | --- |
| **Age** |  | **≤ 60 years** |  | **> 60 years** |  |  |  |
| Regular meat-eaters (reference) | 119 | 1 | 275 | 1 |  |  |  |
| Occasional meat-eaters | 62 | 0.89 (0.65, 1.22) | 185 | 1.07 (0.88, 1.29) |  |  |  |
| Pescatarians | 33 | 0.97 (0.64, 1.47) | 47 | 1.00 (0.72, 1.38) |  |  |  |
| Vegetarians | 48 | 1.31 (0.92, 1.87) | 53 | 1.39 (0.99, 1.96) |  |  | 0.8 |
| **Menopausal status** |  | **Pre-menopausal** |  | **Post-menopausal** |  |  |  |
| Regular meat-eaters (reference) | 38 | 1 | 356 | 1 |  |  |  |
| Occasional meat-eaters | 11 | 0.43 (0.21, 0.86) | 236 | 1.08 (0.91, 1.27) |  |  |  |
| Pescatarians | 17 | 1.17 (0.64, 2.13) | 63 | 0.93 (0.70, 1.24) |  |  |  |
| Vegetarians | 22 | 1.16 (0.68, 1.97) | 79 | 1.37 (1.04, 1.82) |  |  | 0.05 |
| **Physical activity** |  | **< 150 minutes/week** |  | **≥ 150 minutes/week** |  |  |  |
| Regular meat-eaters (reference) | 324 | 1 | 70 | 1 |  |  |  |
| Occasional meat-eaters | 198 | 1.01 (0.84, 1.21) | 49 | 1.03 (0.71, 1.49) |  |  |  |
| Pescatarians | 62 | 1.00 (0.74, 1.34) | 18 | 0.96 (0.56, 1.63) |  |  |  |
| Vegetarians | 81 | 1.44 (1.09, 1.91) | 20 | 1.08 (0.66, 1.80) |  |  | 0.8 |
| **Supplementation** |  | **Yes** |  | **No** |  |  |  |
| Regular meat-eaters (reference) | 180 | 1 | 214 | 1 |  |  |  |
| Occasional meat-eaters | 139 | 1.11 (0.88, 1.39) | 108 | 0.92 (0.73, 1.17) |  |  |  |
| Pescatarians | 49 | 1.03 (0.74, 1.44) | 31 | 0.95 (0.63, 1.42) |  |  |  |
| Vegetarians | 57 | 1.41 (1.02, 1.96) | 44 | 1.32 (0.91, 1.91) |  |  | 0.8 |
| **SES** |  | **Professional/managerial** |  | **Intermediate** |  | **Routine/manual** |  |
| Regular meat-eaters (reference) | 256 | 1 | 51 | 1 | 87 | 1 |  |
| Occasional meat-eaters | 178 | 1.05 (0.86, 1.28) | 38 | 1.13 (0.74, 1.72) | 31 | 0.80 (0.53, 1.21) |  |
| Pescatarians | 59 | 1.02 (0.75, 1.38) | 8 | 0.98 (0.46, 2.01) | 13 | 0.90 (0.49, 1.67) |  |
| Vegetarians | 72 | 1.41 (1.05, 1.91) | 14 | 1.31 (0.71, 2.39) | 15 | 1.23 (0.69, 2.16) | 0.9 |
| **Smoking status** |  | **Current** |  | **Former** |  | **Never** |  |
| Regular meat-eaters (reference) | 58 | 1 | 118 | 1 | 218 | 1 |  |
| Occasional meat-eaters | 31 | 0.90 (0.57, 1.41) | 77 | 1.00 (0.74, 1.34) | 139 | 1.05 (0.85, 1.31) |  |
| Pescatarians | 12 | 0.83 (0.43, 1.61) | 27 | 0.91 (0.59, 1.40) | 41 | 1.09 (0.76, 1.56) |  |
| Vegetarians | 11 | 1.22 (0.59, 2.54) | 33 | 1.23 (0.83, 1.83) | 57 | 1.48 (1.06, 2.06) | 0.9 |

Models were adjusted for ethnicity (white, Asian, black, other), socio-economic status (SES, professional/managerial, intermediate, routine/manual), marital status (married/living as married, separated/divorced, single/widowed), menopausal status (premenopausal, postmenopausal), number of children (continuous), chronic disease prevalence at baseline (yes, no - including stroke, cancer, or diabetes), physical activity in hours per day (continuous), smoking status (current, former, never), alcohol consumption (> 1/week, ≤ 1/week, never), height (continuous), body weight (continuous), and any nutritional supplement use (yes, no). Each stratifying variable was omitted from their adjustment set. HR (95% CI): hazard ratio (95% confidence interval). SES: social economic status.

**Table S7: Risk of hip fracture by diet group with varying restrictions in the UKWCS.**

| **Diet group** | **Cases/subjects** | **Person-years** | **HR (95% CI)** | **p** |
| --- | --- | --- | --- | --- |
| **Adjusted model** | |  |  |  |
| Regular meat-eater (reference) | 394/12221 | 252610 | 1.00 |  |
| Occasional meat-eater | 247/6902 | 145639 | 1.00 (0.85, 1.18) | 0.962 |
| Pescatarian | 80/3377 | 74077 | 0.97 (0.75, 1.26) | 0.818 |
| Vegetarian | 101/3818 | 84042 | 1.33 (1.03, 1.71) | 0.026 |
| **Further adjusted for HRT use** | |  |  |  |
| Regular meat-eater (reference) | 365/11599 | 240994 | 1.00 |  |
| Occasional meat-eater | 228/6530 | 138549 | 0.99 (0.84, 1.18) | 0.949 |
| Pescatarian | 74/3234 | 71156 | 0.96 (0.73, 1.24) | 0.734 |
| Vegetarian | 98/3671 | 80995 | 1.34 (1.06, 1.70) | 0.016 |
| **Excluding subjects with < 5 years of follow-up** | | |  |  |
| Regular meat-eater (reference) | 372/12029 | 252025 | 1.00 |  |
| Occasional meat-eater | 235/6809 | 145359 | 1.02 (0.86, 1.20) | 0.857 |
| Pescatarian | 78/3353 | 73999 | 1.02 (0.78, 1.33) | 0.872 |
| Vegetarian | 97/3796 | 83966 | 1.40 (1.08, 1.81) | 0.011 |
| **Excluding subjects on long-term treatment for illness** |  |  |  |  |
| Regular meat-eater (reference) | 198/8206 | 173067 | 1.00 |  |
| Occasional meat-eater | 148/4739 | 101953 | 1.09 (0.88, 1.37) | 0.424 |
| Pescatarian | 53/2531 | 56141 | 1.08 (0.78, 1.50) | 0.637 |
| Vegetarian | 69/2959 | 65603 | 1.48 (1.07, 2.04) | 0.016 |
| **Further adjusted for baseline fracture prevalence at other sites** | | | |  |
| Regular meat-eater (reference) | 394/12221 | 252610 | 1.00 |  |
| Occasional meat-eater | 247/6902 | 145639 | 1.01 (0.86, 1.19) | 0.971 |
| Pescatarian | 80/3377 | 74077 | 0.97 (0.75, 1.26) | 0.811 |
| Vegetarian | 101/3818 | 84042 | 1.33 (1.03, 1.71) | 0.027 |
| **Vegetarians and vegans separated** | |  |  |  |
| Regular meat-eater (reference) | 394/12221 | 252610 | 1.00 |  |
| Occasional meat-eater | 247/6902 | 145639 | 1.00 (0.85, 1.18) | 0.963 |
| Pescatarian | 80/3377 | 74077 | 0.97 (0.75, 1.26) | 0.817 |
| Vegetarian | 96/3688 | 81250 | 1.35 (1.04, 1.74) | 0.023 |
| Vegan | 5/130 | 2793 | 1.05 (0.40, 2.71) | 0.927 |

The adjusted model was adjusted for ethnicity (white, Asian, black, other), socio-economic status (professional/managerial, intermediate, routine/manual), marital status (married/living as married, separated/divorced, single/widowed), menopausal status (premenopausal, postmenopausal), number of children (continuous), chronic disease prevalence at baseline (yes, no - including stroke, cancer, or diabetes), physical activity in hours per day (continuous), smoking status (current, former, never), alcohol consumption (> 1/week, ≤ 1/week, never), height (continuous), body weight (continuous), and any nutritional supplement use (yes, no). All other models were based on the adjusted model. HR (95% CI): hazard ratio (95% confidence interval). HRT: hormone replacement therapy use at recruitment.

# **Supplementary Methods**

## Diet group classification

The food frequency questionnaire administered at recruitment included questions about consumption of foods and beverages in the form of ‘how often do you eat [specific food or beverage]?’ or similar. Ten responses were possible: 0 ‘never’, 1 ‘< once per month’, 2 ‘1-3 per month’, 3 ‘once per week’, 4 ‘2-4 per week’, 5 ‘5-6 per week’, 6 ‘once per day’, 7 ‘2-3 per day’, 8 ‘4-5 per day’, or 9 ‘6+ per day’. We converted the responses to these questions into daily-based consumption frequencies as follows: 0, 0.02, 0.07, 0.14, 0.4, 0.8, 1, 2.5, 4.5, 6 times per day. Total meat, total fish, total eggs, and total dairy intakes were then calculated in servings per day by summing daily consumptions of relevant items. For example, we summed daily consumptions of beef, pork, lamb, chicken, turkey, bacon, ham, sausages, pies, and offal to derive total meat intake. Similarly, questions on fish intake were summed to calculate total fish intake; and questions on intake of milk, yogurt, cheese, cream, and dairy desserts were summed to calculate total dairy intake in servings per day. Meat, fish, eggs and dairy intakes were then used to classify subjects as regular meat-eaters, occasional meat-eaters, pescatarians, vegetarians, or vegans (Table S2).

## Determining the minimally sufficient adjustment set

Figure S1 shows the Directed Acyclic Graph (DAG) used to inform the multivariable adjustment set for tests of associations between diet group and hip fracture risk. We did not adjust for age at recruitment as this was accounted for by using attained age as the survival time in Cox models. We did not adjust for education due to the high correlation between education and socio-economic status, which was included in the adjustment set. Hormone replacement therapy use was not adjusted for in multivariable adjusted models since it was considered in the definition of menopausal status. Osteoporosis prevalence at recruitment was not adjusted for since there were no confirmed cases at recruitment. Similarly, prevalence of other fracture at baseline was not adjusted for since there were only 10 cases at recruitment.
